# Integrity of the uncinate fasciculus is associated with the onset of bipolar disorder: a 6-year followed-up study

**DOI:** 10.1038/s41398-021-01222-z

**Published:** 2021-02-05

**Authors:** Xiaoyue Li, Weicong Lu, Ruoxi Zhang, Wenjin Zou, Yanling Gao, Kun Chen, Suk-Yu Yau, Robin Shao, Roger S. McIntyre, Guiyun Xu, Kwok-Fai So, Kangguang Lin

**Affiliations:** 1 Department of Affective Disorders, The Affiliated Brain Hospital of Guangzhou Medical University (Guangzhou Huiai Hospital), Guangzhou, China; 2 Laboratory of Emotion and Cognition, The Affiliated Hospital of Guangzhou Medical University (Guangzhou Huiai Hospital), Guangzhou, China; 3 Department of Rehabilitation Sciences, Faculty of Health and Social Sciences, The Hong Kong Polytechnic University, Hong Kong, China; 4 Laboratory of Neuropsychology& Human Neuroscience, University of Hong Kong, Hong Kong, China; 5 Mood Disorders Psychopharmacology Unit, Poul Hansen Family Centre for Depression, University Health Network, Toronto, ON Canada; 6 Department of Psychiatry, University of Toronto, Toronto, ON Canada; 7 Brain and Cognition Discovery Foundation, Toronto, ON Canada; 8 Ministry of Education Joint International Research Laboratory of CNS Regeneration, Jinan University, Guangzhou, China

**Keywords:** Predictive markers, Bipolar disorder

## Abstract

Patients with Bipolar Disorder (BD) are associated with aberrant uncinate fasciculus (UF) that connects amygdala-ventral prefrontal cortex (vPFC) system, but the casual relationship is still uncertain. The research aimed to investigate the integrity of UF among offspring of patients with BD and investigate its potential causal association with subsequent declaration of BD. The fractional anisotropy (FA) and mean diffusivity (MD) of UF were compared in asymptomatic offspring (AO, *n* = 46) and symptomatic offspring (SO, *n* = 45) with a parent with BD, and age-matched healthy controls (HCs, *n* = 35). Logistic regressions were performed to assess the predictive effect of UF integrity on the onset of BD. The three groups did not differ at baseline in terms of FA and MD of the UF. Nine out of 45 SO developed BD over a follow-up period of 6 years, and the right UF FA predicted the onset of BD (*p* = 0.038, OR = 0.212, 95% CI = 0.049–0.917). The ROC curve revealed that the right UF FA predicted BD onset (area-under-curve = 0.859) with sensitivity of 88.9% and specificity of 77.3%. The complementary whole-brain tract-based spatial statistics (TBSS) showed that widespread increases of FA were found in the SO group compared with HCs, but were not associated with the onset of BD. Our data provide evidence supporting the causal relationship between the white matter structural integrity of the amygdala-vPFC system and the onset of BD in genetically at-risk offspring of BD patients.

## Introduction

Bipolar disorder (BD) is a major disabling mental illness, afflicting approximately 1% of the general population and accounting for 0.4% of total DALYs in global burden^1,2^. BD is highly heritable, with heritability of 60.4–85%^3,4^. Extant evidence suggests that there are disruptions in mood regulation networks formed by the prefrontal and subcortical regions^5,6^. Some aberrances were reported in offspring of BD patients, suggesting that they may serve as endophenotypes^7–9^.

Clinical staging models consider the development of BD as comprising several identifiable contiguous stages^10^. Genetically at-risk individuals can be regarded as in the high-risk stage (HR), whereas those with combined genetic risk and subthreshold symptoms can be grouped into the ultra-high-risk stage (UHR)^11,12^, a stage most proximal to the formal full-blown episode. It is worth noting that these hypothetical models need to be tested with prospective longitudinal studies. Available research suggests that up to 25% of genetically at-risk individuals developed into disorders. On the other hand, a larger proportion of genetically high-risk individuals, particularly those without subthreshold symptoms, may have resilience or protective features that prevent them from the onset of disorders^13^. It is crucially important to identify biomarkers capable of predicting the onset of psychiatric disorders^14^. Nevertheless, there have been few longitudinal studies that investigated the objective predictors of BD for genetically at-risk individuals^15–17^.

Amygdala-ventral prefrontal cortex (vPFC) system plays a critical role in emotional processing and mood regulation^18,19^. Abnormal structural changes in the prefrontal cortex and amygdala as well as functional connectivity between the regions have been repeatedly found in patients with BD^6,20^. Moreover, diffusion tensor imaging (DTI) studies found that BD patients had alterations in the white matter (WM) in the amygdala-vPFC system^5^. Uncinate fasciculus (UF) is a main fiber tract that connects the vPFC with amygdala and plays a key role in the emotional regulation circuitry^21^. Patients with BD were found to have decreased fractional anisotropy (FA) of the UF^7,8,22^, a measure that reflects collinearity of longitudinally-aligned fibers and axonal integrity^23^. Furthermore, a recent longitudinal DTI study by Weathers^24^ reported that adolescents/young adults with BD had lower time-related expansion of FA in the UF, suggesting that its abnormal development may be related to BD pathology. Decreased FA in the UF was found in the first-degree relatives besides BD patients^8^.

Given the above considerations, we hypothesized that the integrity of the WM of the UF may be altered in the genetically at-risk offspring of parents with BD and could serve as biomarkers capable of predicting the onset of mood disorders for the at-risk individuals. To this end, we first did cross-sectional comparisons in the integrity (i.e. FA and mean diffusivity (MD)) of the UF between symptomatic and asymptomatic offspring of parents with BD and health controls, respectively. We then investigated whether baseline integrity measures of the UF could predict the prognosis of a cohort of offspring of parents with BD over a follow-up period of 6 years. As a complementary analysis, we additionally conducted a whole-brain tract-based spatial statistics (TBSS) between the groups.

## Method

### Participants

The data were derived from the recognition and early intervention on prodromal bipolar disorder (REI-PBD) project^25^ that was launched in 2013, in which we followed-up a cohort of offspring of parents with BD. The project was approved by the Institutional Review Board of Guangzhou Brain Hospital. All participants and their guardians (if aged under 18 years) provided written informed consent.

Participants were screened for lifetime Axis I disorders at baseline interview using The Schedule for Affective Disorders and Schizophrenia for School-aged Children−Present and Lifetime versions (K-SADS-PL) or the Structured Clinical Interview for DSM-IV-TR Axis I Disorders, Research Version, Patient Edition (SCID-I/P) (if participants aged beyond 18 years). Hamilton depression rating scale (HAMD), Hamilton anxiety rating scale (HAMA), and the young mania rating scale (YMRS) were applied for evaluation of depressive symptoms and hypomanic symptoms, respectively. The follow-up assessments were prospectively performed at 3 months and annually. Age-matched health controls (HCs) were recruited through self-referred, advertisement, or word-by-mouth in the same manner as the offspring of parents with bipolar disorder.

Based on whether they manifested subthreshold mood symptoms, offspring of parents of BD were further divided into groups of asymptomatic and symptomatic offspring (AO and SO, respectively. Subthreshold mood symptoms were defined as below: (i) two or more hypomania symptoms lasting at least 4 days, but not meeting the criteria of hypomania defined by DSM-IV; (ii) two or more major depressive symptoms lasting at least 1 week, but not meeting the criteria of major depressive episode defined by DSM-IV; (iii) one or more attenuated psychotic symptoms (i.e., ideas of reference, odd ideas, odd beliefs, unusual perceptual, experiences, bizarre thoughts or speech, grandiosity, suspicious ideas, paranoid ideas, odd mannerisms, hallucinations, disorganized/catatonic behaviors) lasting at least 10 min for each symptom, with 2–7 times per week for at least 3 months; or (iv) two or more DSM-IV defined hyperactivity and impulsivity symptoms/signs observed by teachers, peers, and/or parents^26^. Offspring (*n* = 91) of parents with BD and HCs (*n* = 35) completed at least one scheduled followed-up assessment. Among the 91 bipolar offspring, 46 were identified as AO at baseline and 45 SO. During this up-to-6-year longitudinal follow-up study, we applied the DSM-IV diagnostic criteria when confirming conversion cases. Most conversion cases were confirmed by face-to-face psychiatric interview, and a few cases were confirmed by medical records and telephone interview.

### MRI Acquisition and Preprocessing

All data of participants at baseline were acquired on Philips 3.0 T MRI scanner equipped with a 8-channel SENSE head-coil. 32 non-collinear (b value = 1000 s/mm^2^)-direction diffusion images and a no-diffusion weighting (b0) were collected using echo-planar imaging sequence with the following parameters: field of view (FOV) = 256 × 256, TR/TE = 10086/91 ms, 2 mm slice thickness with no gap, matrix = 128 × 128, voxel size = 2 × 2 × 2 mm^3^. All images were corrected for eddy current distortion and head movement by registering diffusion-weighted images to b0 and adjusting rotations of b-matrix using FMRIB’s diffusion toolbox (FDT), part of FMRIB Software Library (FSL). FA and MD were calculated by DTIfit (https://fsl.fmrib.ox.ac.uk/fsl/fslwiki/FDT).

### Region-of-interest analysis and Tract-based spatial statistics (TBSS)

Tract ROI (UF) was created from probabilistic tract map based on JHU ICBM-DTI-81 atlas using fsl maths command in FSL^27–29^. Mean FA and MD of bilateral UF were calculated for each participant. Tract-based spatial statistics (TBSS) analysis was carried out for voxel-wise statistical analysis of FA^30^. After aligning each participant’s FA data into 1 × 1 × 1 mm^3^ standard Montreal Neurological Institute (MNI152) space using non-linear registration^31,32^, the mean FA was created and thinned to generate a mean FA skeleton which represented the centers of all the tracts derived from all participants. Then, each aligned FA was projected onto the skeleton. The above procedure was also applied for the calculation of MD (https://fsl.fmrib.ox.ac.uk/fsl/fslwiki/TBSS/UserGuide). Non-parametric permutation for voxel-wise statistics inference provided by FSL’s randomization procedure was conducted to detect the group difference. Taking age and gender as covariates, threshold-free cluster enhancement (TFCE) was applied for multiple comparisons, with 5000 permutations for each contrast. The anatomical location of significant clusters (*P* < 0.05) was identified by Johns Hopkins University (JHU) ICBM-DTI-81 white-matter labels atlas^27^.

### Statistical analysis

Demographic and clinical characteristics were analyzed using ANOVA and non-parametric tests (Mann-Whitney or Kruskal-Wallis test). Primary ROI analyses were performed using general linear models (GLM) in SPSS that separately included FA or MD as the dependent variable, group (AO, SO, or HC) as a fixed factor, and age and gender as covariates. Logistic regression was used to examine whether bilateral UF diffusion values (FA and MD) contributed to the prediction of subsequent onset of BD over the follow-up period. Diffusion values were converted to standardized scores (z-scores). Significant variables identified by the logistic regression models were used to build a receiver operating characteristic (ROC) curve and its area under the curve (AUC). All analyses were two-tailed and *P-value* was set at 0.05. Statistical analyses were carried out using SPSS version 24.0.

## Results

### Demographic and clinical characteristics

As shown in Table 1, the three groups were matched on age, gender, and handedness. The SO group scored significantly higher in the HAMD, HAMA, and YMRS than the AO group and HCs (*p* < 0.05). No significant difference in the HAMD, HAMA, orYMRS was found between the AO and the HCs (*p* > 0.05). Over the 6-year follow-up, 9 SO developed BD, and none of the AO group or HCs developed BD.

**Table 1:** Demographics and clinical characteristics among AO, SO, and HCs.

	AO	SO	HCs	Statistic	*P*-value
	*N* = 46	*N* = 45	*N* = 35		
	M (SD) or Total	M (SD) or Total	M (SD) or Total		
Demographic information					
Age	17.00 (4.70)	17.62 (5.37)	15.09 (3.74)	*F* = 3.00	0.053
Sex (female)	27	27	22	*χ*^*2*^ = 0.147	0.929
Handedness (right)	45	44	35	*χ*^*2*^ = 0.782	0.676
Clinical measure					
HAMD	0.43 (0.91)	7.96 (9.05)	0.43 (1.09)	*F* = 27.33	<**0.001**
HAMA	0.48 (0.89)	5.89 (7.42)	0.60 (1.56)	*F* = 20.11	<**0.001**
YMRS	0.52 (1.44)	2.00 (3.04)	0.14 (0.85)	*F* = 9.49	<**0.001**

F = ANOVA test statistical value; *χ*^2^ = chi-squared test statistical value; AO: asymptomatic offspring of patients with BD; SO: symptomatic offspring of patients with BD; *HCs* health controls; HAMD: Hamilton Depression Rating Scale; HAMA: Hamilton Anxiety Scale; *YMRS* Young Manic Rating Scale; *M* = mean; *SD* = standard deviation.

Bold values indicate statistical significance.

### FA and MD comparisons between groups

We compared the FA of the left and right UF among the three groups and did not find any statistical significance (F = 0.821, *p* = 0.443; F = 0.354, *p* = 0.703, respectively). No significant difference in the MD of the left and right UF was found among the three groups (F = 0.995, *p* = 0.373; F = 0.198, *p* = 0.820, respectively).

### Predictors for onset of BD

**Fig. 1:**
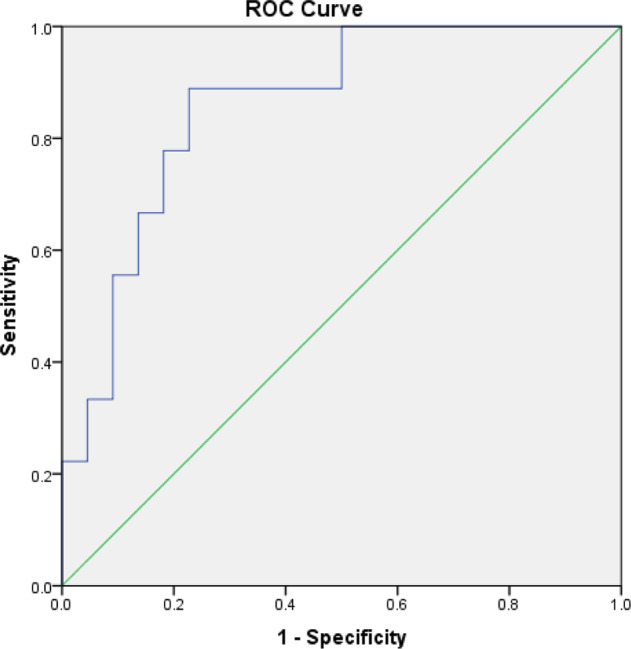
Receiver operating characteristic (ROC) curve for right UF FA in BD development. Area under the ROC curve = 0.859 (95% CI 0.723–0.994).

As shown in Table 2, univariate logistic regression analyses showed that baseline severity of anxiety (measured by HAMA) and the integrity of the right UF (measured by FA) could predict the onset of BD (*p* = 0.047, OR = 1.114, 95% CI = 1.001–1.238; *p* = 0.021, OR = 0.179, 95% CI = 0.041–0.775, respectively). Thecorrelationanalysisshowed that the HAMA scores were significantly correlated withthe right UFFA (*r* = 0.379, *P* = 0.036). After controlling for HAMA, the predictive capacity of the right UF FA for the onset of BD did not change, with the OR value slightly increased from 0.179 to 0.212 (*p* = 0.038, OR = 0.212, 95% CI = 0.049–0.917). The corresponding ROC curve for the right UF FA is showed in Fig. 1. The AUC was 0.859 (95% CI 0.723–0.994, *P* = 0.002), with 88.9% sensitivity and 77.3% specificity.

**Table 2:** Baseline characteristics related to subsequent onset of BD.

Characteristics	Univariate			Multivariate		
	OR	95% CI	*P*-value	OR	95% CI	*P*-value
Age	1.032	0.899–10184	0.658	—	—	
Gender	0.8	0.168–3.799	0.779	—	—	
HAMD	1.055	0.973–1.144	0.199	—	—	
HAMA	1.114	1.001–1.238	**0.047**	1.088	0.968–1.224	0.157
YMRS	1.24	0.978–1.571	0.075	—	—	
Left UF FA	0.366	0.13–1.033	0.058	—	—	
Right UF FA	0.179	0.041–0.775	**0.021**	0.212	0.049–0.917	**0.038**
Left UF MD	0.797	0.337–1.885	0.605	—	—	
Right UF MD	1.241	0.564–2.726	0.592	—	—	

Left UF *FA* = Fractional anisotropy of left uncinate fasciculus; Right UF *FA* = Fractional anisotropy of right uncinate fasciculus; Left UF *MD* = Mean diffusivity of left uncinate fasciculus; Right UF *MD* = Mean diffusivity of right uncinate fasciculus; *OR* = odds ratio; *SD* = standard deviation; *CI* = confidence interval.

Bold values indicate statistical significance.

### Complementary whole-brain TBSS analysis

**Fig. 2:**
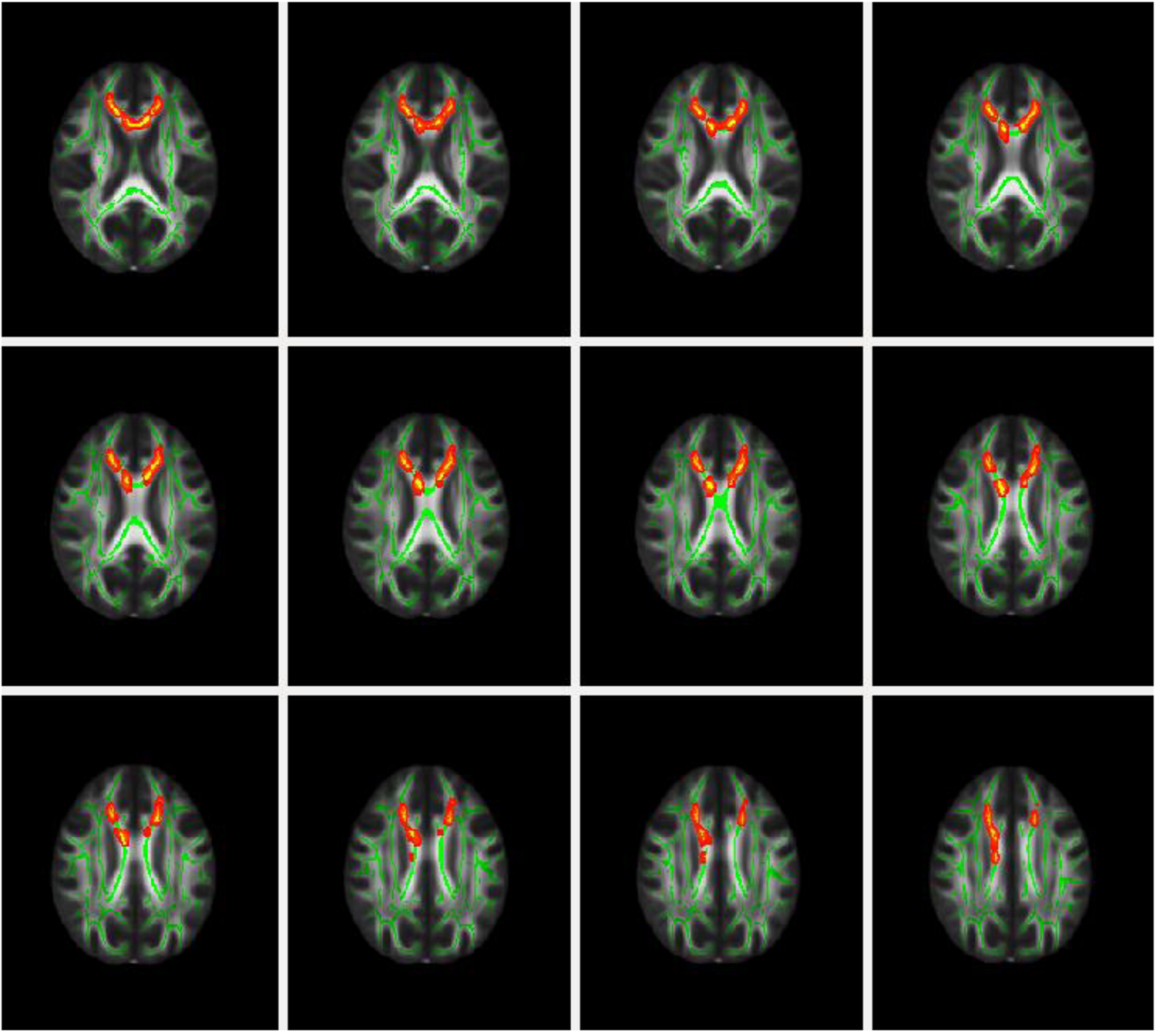
TBSS between symptomatic offspring of patients with BD and healthy controls. Axial rendering showing significant difference in symptomatic offspring of patients with BD (SO) compared with health controls (HCs) obtained from Tract-based Spatial Statistic (TBSS) analysis. Significant increased FA in UHR (*P* < 0.05, TFCE) was represented by red and yellow color in genu of corpus callosum, body of corpus callosum, left anterior corona radiata, right anterior corona radiata, left superior corona radiata, and right superior corona radiata. Mean FA skeleton of SO and HCs individuals (green color) was overlaid on MNI FA 1 mm skeleton template. The result was thickened with “tbss-fill” command provided by FSL. The image follows radiological convention.

Whole-brain TBSS analysis showed that the SO group displayed higher FA than HCs in a cluster including the genu and body of corpus callosum, bilateral anterior corona radiate, and bilateral superior corona radiata (Fig. 2, Table 3). No significant difference in FA was found in the AO group versus HCs (*p* > 0.05). To avoid alignment problem for younger participates between the SO and HCs, supplementary TBSS analyses were conducted by excluding a few participants younger than 10 years old, and the results did not change significantly.

**Table 3:** Anatomical locations of significant voxelwise analysis.

voxels	COG X	COG Y	COG Z	WMT
				Genu of corpus callosum
				Body of corpus callosum
				Anterior corona radiata R
				Anterior corona radiata L
				Superior corona radiata R
2215	86.5	149	91.7	Superior corona radiata L

*COG* = Centre Of Gravity; *WMT* = white matter tract; *R* = right; *L* = left

## Discussion

To our knowledge, this is the first longitudinal DTI study that investigated the associations between the diffusion measures of the UF and the long-term clinical outcomes in the offspring of parents with BD. We found that the right UF FA at baseline had 88.9% sensitivity and 77.3% specificity in predicting the onset of BD over a 6-year follow-up period. This finding supports our white-matter predictive model over the prospective development of BD.

The amygdala-vPFC system, connected by the UF, was involved in voluntary subprocesses and operated by a feedback mechanism^5,33^. Lower UF FA which reflects axonal loss and demyelination of WM tract^34^ may result in abnormal bidirectional fronto-subcortical connectivity and aberrant “feedback” connectivity that lead to insufficient prefrontal inhibitory control over subcortical structures^33,35,36^. Moreover, a recent DTI study showed that integrity of the UF (measured by FA) was linked to amygdale processing of fearful faces^37^. Furthermore, the right UF integrity was positively correlated with performance in encoding and decoding of facial emotional expressions which were related to social behavior and emotional functioning^38^. Taken together, we thus speculate that the abnormal development of the UF over time is associated with the onset of BD. On the other hand, we did not find significant changes in the UF FA in the SO group versus HCs at baseline, consistent with other familiar studies^39,40^. One possibility may be that the change of UF FA is subtle at baseline so that our sample size did not have the statistical power to detect the difference.

In the whole-brain TBSS exploratory analysis, increased FA was found in the SO group when compared to HCs in a cluster including the genu and body of corpus callosum, bilateral anterior corona radiate, and bilateral superior corona radiate. However, the changes were not related to the onset of BD (data not shown). An increased FA may indicate excessive myelination^34^, probably reflective of overdeveloped pathways to compensate for pathological processes^41,42^. The anterior corpus callosum carried left and right ventral prefrontal cortex connections engaging in cognitive processing and emotional regulation^43,44^. Abnormalities in the corona radiata, which connects brainstem with prefrontal regions including the ventrolateral prefrontal cortex (VLPFC) and dorsolateral prefrontal cortex (DLPFC)^45,46^, are repeatedly reported in patients with BD^47,48^. This finding of increased FA in the WM might suggest signs of compensatory reactions to the subthreshold mood symptoms manifested in the bipolar offspring as we previously observed^25,26^.

There are some limitations that should be mentioned when interpreting the findings. First, the method of the whole-tract approach does not take into account the intra-tract characteristics as tissue characteristics may differ along a WM tract^49^. Existing conceptual framework has implicated the UF tract as being divided into three prefrontal stems in which the medial stem that extends from BA25 is more implicated in mood regulation^50^. Future studies should investigate the subregions of UF. Second, our sample size is small and the findings might be only generalizable to genetically at-risk bipolar offspring. Third, the scope of this study was limited to the integrity of UF at baseline in predicting the onset of BD. Future studies are warranted for illustrating the trajectory of change of UF to clarify the UF effect on the development of BD.

## Conclusion

Bipolar disorder is highly heritable, yet the long-term clinical outcomes of genetically at-risk offspring are largely heterogeneous. In this 6-year longitudinal study, our data suggest that the integrity of the right UF can predict the onset of BD in a cohort of bipolar offspring. Future prediction models for bipolar disorder should attempt to replicate and extend our findings.

## References

[1] Ferrari AJ (2016). The prevalence and burden of bipolar disorder: findings from the Global Burden of Disease Study 2013. Bipolar Disord..

[2] Grande I, Berk M, Birmaher B, Vieta E (2016). Bipolar disorder. Lancet.

[3] McGuffin P (2003). The heritability of bipolar affective disorder and the genetic relationship to unipolar depression. Arch. Gen. Psychiatry.

[4] Johansson V, Kuja-Halkola R, Cannon TD, Hultman CM, Hedman AM (2019). A population-based heritability estimate of bipolar disorder - in a Swedish twin sample. Psychiatry Res..

[5] de Zwarte SM, Johnston JA, Cox Lippard ET, Blumberg HP (2014). Frontotemporal white matter in adolescents with, and at-risk for, bipolar disorder. J. Clin. Med..

[6] Lu X (2019). Structural imaging biomarkers for bipolar disorder: meta-analyses of whole-brain voxel-based morphometry studies. Depression Anxiety.

[7] Sarıçiçek A (2016). Abnormal white matter integrity as a structural endophenotype for bipolar disorder. Psychological Med..

[8] Mahapatra A, Khandelwal SK, Sharan P, Garg A, Mishra NK (2017). Diffusion tensor imaging tractography study in bipolar disorder patients compared to first-degree relatives and healthy controls. Psychiatry Clin. Neurosci..

[9] Hanford LC, Hall GB, Minuzzi L, Sassi RB (2016). Gray matter volumes in symptomatic and asymptomatic offspring of parents diagnosed with bipolar disorder. Eur. Child Adolesc. Psychiatry.

[10] Scott J, Henry C (2018). [The staging model (or evolutionary stages) applied to bipolar disorder]. L’Encephale.

[11] Frank E, Nimgaonkar VL, Phillips ML, Kupfer DJ (2015). All the world’s a (clinical) stage: rethinking bipolar disorder from a longitudinal perspective. Mol. Psychiatry.

[12] Scott J (2013). Clinical staging in psychiatry: a cross-cutting model of diagnosis with heuristic and practical value. Br. J. Psychiatry.

[13] Raouna A, Osam CS, MacBeth A (2018). Clinical staging model in offspring of parents with bipolar disorder: a systematic review. Bipolar Disord..

[14] Frangou, S. Neuroimaging markers of risk, disease expression, and resilience to bipolar disorder. *Curr. Psychiatry Rep.* **21**, 10.1007/s11920-019-1039-7 (2019). 10.1007/s11920-019-1039-7 31161278

[15] Nimarko AF, Garrett AS, Carlson GA, Singh MK (2019). Neural correlates of emotion processing predict resilience in youth at familial risk for mood disorders. Dev. Psychopathol..

[16] Zhang, W. et al. Individual prediction of symptomatic converters in youth offspring of bipolar parents using proton magnetic resonance spectroscopy. *Eur Child Adolesc Psychiatry*, 10.1007/s00787-020-01483-x (2020). 10.1007/s00787-020-01483-x 32008167

[17] Brietzke E (2012). Towards a multifactorial approach for prediction of bipolar disorder in at risk populations. J. Affect Disord..

[18] Blond BN, Fredericks CA, Blumberg HP (2012). Functional neuroanatomy of bipolar disorder: structure, function, and connectivity in an amygdala-anterior paralimbic neural system. Bipolar Disord..

[19] Strakowski SM (2012). The functional neuroanatomy of bipolar disorder: a consensus model. Bipolar Disord..

[20] Pfeifer JC, Welge J, Strakowski SM, Adler CM, DelBello MP (2008). Meta-analysis of amygdala volumes in children and adolescents with bipolar disorder. J. Am. Acad. Child Adolesc. Psychiatry.

[21] Von Der Heide RJ, Skipper LM, Klobusicky E, Olson IR (2013). Dissecting the uncinate fasciculus: disorders, controversies and a hypothesis. Brain.

[22] Deng F (2018). Abnormal segments of right uncinate fasciculus and left anterior thalamic radiation in major and bipolar depression. Prog. Neuropsychopharmacol. Biol. Psychiatry.

[23] Soares JM, Marques P, Alves V, Sousa N (2013). A hitchhiker’s guide to diffusion tensor imaging. Front Neurosci..

[24] Weathers J (2018). Longitudinal diffusion tensor imaging study of adolescents and young adults with bipolar disorder. J. Am. Acad. Child Adolesc. Psychiatry.

[25] Lin K (2015). A multi-dimensional and integrative approach to examining the high-risk and ultra-high-risk stages of bipolar disorder. EbioMedicine.

[26] Lin K (2018). Resting-state fMRI signals in offspring of parents with bipolar disorder at the high-risk and ultra-high-risk stages and their relations with cognitive function. J. Psychiatr. Res..

[27] Wakana S (2007). Reproducibility of quantitative tractography methods applied to cerebral white matter. Neuroimage.

[28] Hua K (2008). Tract probability maps in stereotaxic spaces: analyses of white matter anatomy and tract-specific quantification. Neuroimage.

[29] Mori, S., Wakana, S., Van Zijl, P. C. & Nagae-Poetscher, L. *MRI atlas of human white matter*. (Elsevier, 2005). 10.1148/radiol.2301021640 14645885

[30] Smith SM (2006). Tract-based spatial statistics: voxelwise analysis of multi-subject diffusion data. Neuroimage.

[31] Rueckert D (1999). Nonrigid registration using free-form deformations: application to breast MR images. IEEE Trans. Med. imaging.

[32] Hutton, J. S., Dudley, J., Horowitz-Kraus, T., DeWitt, T. & Holland, S. K. Associations between screen-based media use and brain white matter integrity in preschool-aged children. *JAMA Pediatr.*, e193869, 10.1001/jamapediatrics.2019.3869 (2019). 10.1001/jamapediatrics.2019.3869 PMC6830442 31682712

[33] Phillips ML, Ladouceur CD, Drevets WC (2008). A neural model of voluntary and automatic emotion regulation: implications for understanding the pathophysiology and neurodevelopment of bipolar disorder. Mol. Psychiatry.

[34] Marlinge E, Bellivier F, Houenou J (2014). White matter alterations in bipolar disorder: potential for drug discovery and development. Bipolar Disord..

[35] Versace A (2008). Elevated left and reduced right orbitomedial prefrontal fractional anisotropy in adults with bipolar disorder revealed by tract-based spatial statistics. Arch. Gen. Psychiatry.

[36] Strakowski SM, Delbello MP, Adler CM (2005). The functional neuroanatomy of bipolar disorder: a review of neuroimaging findings. Mol. Psychiatry.

[37] Hein TC (2018). Amygdala habituation and uncinate fasciculus connectivity in adolescence: a multi-modal approach. Neuroimage.

[38] Coad, B. M. et al. Structural connections support emotional connections: uncinate Fasciculus microstructure is related to the ability to decode facial emotion expressions. *Neuropsychologia*, 10.1016/j.neuropsychologia.2017.11.006 (2017). 10.1016/j.neuropsychologia.2017.11.006 PMC7534036 29122609

[39] Sprooten E (2016). A comprehensive tractography study of patients with bipolar disorder and their unaffected siblings. Hum. Brain Mapp..

[40] Sprooten E (2013). Reduced white matter integrity in sibling pairs discordant for bipolar disorder. Am. J. Psychiatry.

[41] Roybal DJ (2015). Widespread white matter tract aberrations in youth with familial risk for bipolar disorder. Psychiatry Res..

[42] Beaulieu C (2002). The basis of anisotropic water diffusion in the nervous system: a technical review. NMR Biomed..

[43] Poletti S (2015). Cognitive performances associate with measures of white matter integrity in bipolar disorder. J. Affect Disord..

[44] Bellani M (2009). DTI studies of corpus callosum in bipolar disorder. Biochem Soc. Trans..

[45] Wakana S, Jiang H, Nagae-Poetscher LM, van Zijl PC, Mori S (2004). Fiber tract-based atlas of human white matter anatomy. Radiology.

[46] Catani M, Howard RJ, Pajevic S, Jones DK (2002). Virtual in vivo interactive dissection of white matter fasciculi in the human brain. Neuroimage.

[47] Pavuluri MN (2009). Diffusion tensor imaging study of white matter fiber tracts in pediatric bipolar disorder and attention-deficit/hyperactivity disorder. Biol. Psychiatry.

[48] Linke JO (2020). White matter microstructure in youth with and at risk for bipolar disorder. Bipolar Disord..

[49] Johnson RT (2014). Diffusion properties of major white matter tracts in young, typically developing children. Neuroimage.

[50] Bhatia KD, Henderson LA, Hsu E, Yim M (2018). Reduced integrity of the uncinate fasciculus and cingulum in depression: a stem-by-stem analysis. J. Affect Disord..

